# Involving underrepresented groups: How unpaid carers influenced our data analysis

**DOI:** 10.23889/ijpds.v8i2.2253

**Published:** 2023-09-14

**Authors:** Anna Lawrence-Jones, Jodie Chan, Evgeniy Galimov, Roberto Fernandez Crespo, Sandeep Prashar, Clare McCrudden, Thomas Lewis, Shereen Aloysius, Jacob Oguntimehin, Rachel McCarthy, Helena Gavrielides, Melanie Leis, Alex Bottle, Matthew Chisambi

**Affiliations:** 1 Institute of Global Health Innovation, Imperial College London, London, United Kingdom; 2 Imperial College Health Partners, London, United Kingdom; 3 Research Governance and Integrity Team, Imperial College London, London, United Kingdom; 4 School of Public Health, Imperial College London, London, United Kingdom

## Objectives

The recent census found that five million people in England and Wales provide unpaid care. With social services struggling, unpaid carers face increasing pressure. The North West London Networked Data Lab aimed to understand unpaid carers’ needs, health issues, and care pathways through public involvement and analysis of linked datasets.

## Method

We used the Discover dataset containing primary, secondary, mental health, and social care data of 2.5 million North West Londoners to explore our aims. To ensure the questions asked of the data mattered locally, we interviewed five unpaid carers to understand the issues they faced. One carer worked more closely with the data analyst to define the questions. The interim results were presented to a diverse group of unpaid carers to see whether anything resonated with them, surprised them, or required further research. The group also helped develop an engaging and accessible infographic to communicate our findings.

## Results

The unpaid carer cohort in our dataset were, on average, older females from deprived areas, highlighting gender and socioeconomic inequities in caring responsibilities. Unpaid carers had a higher prevalence of long-term conditions before they were identified as a carer (e.g. hypertension, depression, anxiety and diabetes) and were more likely to use healthcare services than non-carers. Through speaking to unpaid carers, we learned that many hadn’t identified as a carer or mentioned it to their GP for many years. In fact, they had only had their carer status recorded after visiting their GP for an issue linked to their caring responsibilities. Our public involvement helped to highlight a major limitation of the data, particularly as men are less likely to interact with their GPs.

## Conclusion

Our analysis found unpaid carers were more likely to have certain conditions and more likely to have multiple long-term conditions. Public involvement was critical in making sense of these findings and identifying policy and practice recommendations. Giving people a meaningful voice in population data research can also build public trust.

